# The working mechanisms of an environmentally tailored physical activity intervention for older adults: a randomized controlled trial

**DOI:** 10.1186/1479-5868-6-83

**Published:** 2009-12-08

**Authors:** Maartje M van Stralen, Hein de Vries, Aart N Mudde, Catherine Bolman, Lilian Lechner

**Affiliations:** 1 Open University of the Netherlands, Department of Psychology, PO Box 2960, 6401 DL, Heerlen, The Netherlands; 2 VU University Medical Center, EMGO Institute for Public Health and Care Research, Van der Boechorststraat 7, 1081 BT Amsterdam, The Netherlands; 3 Maastricht University, Department of Health Promotion, PO Box 616, 6200 MD, Maastricht, The Netherlands

## Abstract

**Background:**

The aim of this study was to explore the working mechanisms of a computer tailored physical activity intervention for older adults with environmental information compared to a basic tailored intervention without environmental information.

**Method:**

A clustered randomized controlled trial with two computer tailored interventions and a no-intervention control group was conducted among 1971 adults aged ≥ 50. The two tailored interventions were developed using Intervention Mapping and consisted of three tailored letters delivered over a four-month period. The basic tailored intervention targeted psychosocial determinants alone, while the environmentally tailored intervention additionally targeted environmental determinants, by providing tailored environmental information. Study outcomes were collected with questionnaires at baseline, three and six months and comprised total physical activity (days/week), walking (min/week), cycling (min/week), sports (min/week), environmental perceptions and use and appreciation of the interventions.

**Results:**

Mediation analyses showed that changes in cycling, sports and total physical activity behaviour induced by the environmentally tailored intervention were mediated by changes in environmental perceptions. Changes in environmental perceptions did not mediate the effect of the basic tailored intervention on behaviour. Compared with the basic tailored intervention, the environmentally tailored intervention significantly improved cycling behaviour (τ = 30.2). Additionally, the tailored letters of the environmentally tailored intervention were better appreciated and used, although these differences did not mediate the intervention effect.

**Discussion:**

This study gave some first indications of the relevance of environmental perceptions as a determinant of changing physical activity behaviours and the potential effectiveness of providing environmental information as an intervention strategy aimed at enhancing physical activity behaviour among older adults.

## Background

Sufficient physical activity [PA], defined as meeting the international recommendation of 30 minutes of moderate PA a day for at least five days of the week [1], has been associated with a decreased risk of health problems that are particularly salient among older adults, such as cardiovascular diseases, obesity, type 2 diabetes and functional and cognitive dysfunction [2-4] Despite these known physical and psychological health benefits, in the Netherlands, like in most western countries, only 60% of the general population meets the international PA recommendation [5,6]. The number of those complying with the guideline declines with age to less then 50% among older adults[5,6]. In order to increase PA behaviour among older adults, there is a need for low-cost (e.g. computer-tailored) PA interventions that are effective for and applicable to older adults.

Research has shown that physical activity behaviour of older adults can be effectively changed through intervention programs [7-14]. Computer tailored interventions, in which computer technology adapts health information to the specific needs and characteristics of a person [15,16], is a low-cost strategy that has the potential to reach a large population. Computer tailoring has shown promising effects in different health promotion programs [17,18], also among older adults [10,11,13]. However, only a few of these studies have analyzed the working mechanisms through which the interventions exerted its effect. Knowledge of these mechanisms- comprises the analysis of mediators of intervention effects- can provide information for future intervention developers about which intervention strategies are effective in changing behaviours. The aim of this study was to examine the working mechanisms of two computer tailored PA interventions for older adults.

So far, most health promotion intervention studies have addressed solely psychosocial mediators in order to change behaviour. For example, Brassington et al. found in a telephone-based exercise counselling study among 102 older adults, that changes in self-efficacy and fitness outcome realizations were significant predictors of adherence to an exercise program [19]. In order to increase the effectiveness of health promotion interventions, recent theoretical models and empirical research have acknowledged that in addition to targeting individual psychosocial mediators, interventions should also target environmental mediators [20-22]. Several observational studies gave indications that interventions aiming at achieving realistic perceptions of PA possibilities in the immediate environment of the target group might be important in changing PA behaviour [23-25], especially among older adults [26,27]. This was confirmed by Ferney et al. [28] who recently conducted a randomized controlled trial among middle-aged adults. The authors found that participants who had access to an environmentally tailored PA website reported significantly higher PA levels and higher levels of engagement than participants who had access to a non-tailored motivational- information website [28]. This study, however, made the comparison with a non-tailored control group, precluding conclusions on the added effects of providing environmental information.

This paper addresses recent theoretical developments by conducting an in-depth analysis on the working mechanisms of adding environmental information to a computer tailored PA intervention. A randomized controlled trial was conducted in which the efficacy of an environmentally tailored PA intervention (i.e. environmental plus motivational-focused intervention) was compared with a basic tailored intervention (i.e. motivational-focused intervention) and a no-intervention control group [29]. In an earlier publication, we reported the analyses of the efficacy of both interventions, in which medium effect sizes were found for both interventions in changing total weekly days of PA behaviour (ES = 0.35 for environmentally tailored intervention; ES = 0.30 for basic tailored intervention) compared with the no-intervention control condition [30]. The aim of the present paper is to explore the effect of adding environmental information to a computer tailored intervention in two ways. First, we examined whether the working mechanisms (i.e. mediators) of an environmentally tailored intervention differed from a basic tailored intervention without environmental information. Since providing environmental information was expected to change environmental perceptions, these changes were tested as a mediator of the intervention effect. It was hypothesized that environmental perceptions would mediate the intervention effect of the environmentally tailored intervention condition only. Since the additional environmental information provided information about walking, cycling and sports opportunities, differences in efficacy and mediated effects between the two interventions on these activities were most likely to occur and therefore expected. Second, we examined whether providing environmental information increased the use and appreciation of the intervention, and whether these increases mediated the intervention effect. Since the tailored letters of the environmentally tailored intervention contained more personalized and comprehensive information about PA possibilities in the participants' environment, it was hypothesized that in agreement with the study of Ferney et al. [28], the tailored letters of the environmentally tailored intervention were evaluated more positively and were used more often than the letters of the basic tailored intervention. In addition, it was expected that these differences in appreciation would partly mediate the differences in intervention effects.

## Method

For the purpose of the study, an RCT was conducted, which was registered at the Dutch Trial Register (NTR920) and approved by the Medical Ethics Committee of Maastricht University and the University Hospital Maastricht.

### Participants and procedure

**Figure 1:**
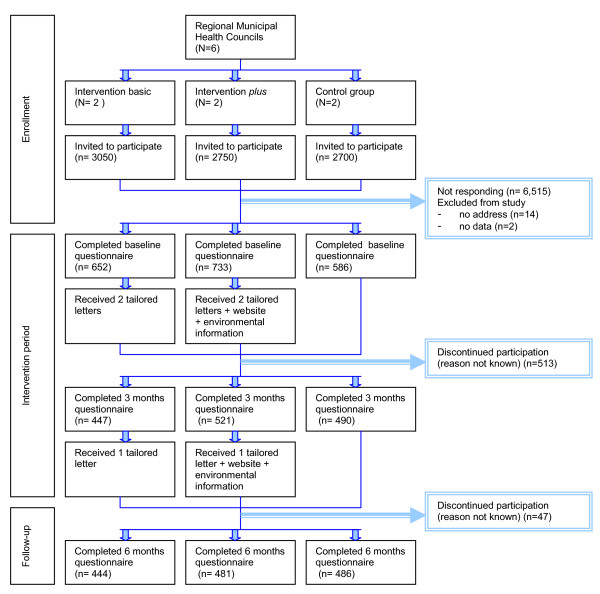
**Flow diagram of the selection and enrolment of the study participants**.

The study procedure, including the selection and enrolment of participants and the distribution of the questionnaires and interventions are shown in Figure 1. In 2007, all Dutch Regional Municipal Health Councils (MHCs) (n = 39) were invited to participate in the program. Nine MHCs agreed to participate, after which six MHCs were randomly selected and assigned to one of the three research arms: (1) *basic tailored intervention*, a motivational focused computer tailored intervention targeting psychosocial determinants; (2) *intervention plus*, an environmentally tailored intervention targeting environmental determinants in addition to the tailored feedback of the basic intervention, or (3) *wait list control group*. Randomization was stratified by urbanization level (rural vs urban (i.e. situated in the urban area of the Netherlands ("Randstad")). In total, 8,500 adults aged ≥ 50 years were invited to participate using an invitation letter that was accompanied by a baseline questionnaire and informed consent form. At the start of participation, study participants were eligible to win two city trips or several gift vouchers. At baseline, 1,971 older adults returned the questionnaire (response rate: 23%). Baseline measurement lasted from March to June 2007. Data analysis were conducted in 2008.

### Intervention

The interventions were developed according to the Intervention Mapping protocol [31], a six-step protocol that facilitates a stepwise process for theory-and evidence-based development of health promotion interventions. The determinants targeted by the intervention programs were based on a Delphi study [32] and a literature review [33]. Theory based methods and strategies were derived from theoretical models such as the such as the I-Change Model [34], the Health Action Process Approach[35], the Precaution Adoption Process Model[36], the Self-regulation theory [37], and the Self-determination theory[38]. The content of the interventions including the targeted determinants, theoretical methods and intervention strategies aimed at targeting the selected determinants has been described in more detail elsewhere [29], and is shortly described below. Since print-delivered computer tailored interventions were found to be more effective in changing PA maintenance of older adults when compared to telephone tailored interventions [7], two print tailored interventions were developed, including paper and pencil questionnaires and printed letters.

*Basic tailored intervention *participants (n = 654) received three tailored letters with personalized physical activity advice. The first and second tailored letters were based on the personal data gathered at baseline and were sent two weeks and two months after baseline respectively (see Figure 1). The third letter was sent two weeks after receiving the three months questionnaire and was based on data gathered at baseline and three months, and addressed the changes the participant had undertaken in these three months. The intervention tried to influence awareness, initiation and maintenance of physical activity by targeting theoretical concepts that underlie each specific phase by addressing pre-motivational constructs (i.e. awareness, knowledge) [32-34,36], motivational constructs (i.e. attitude, social influence, self-efficacy, intention, and intrinsic motivation) [32-35,38] and post-motivational constructs (i.e. commitment, strategic planning, action planning and coping planning)[32-35,37]. The letters comprised between the 3 and 11 pages, depending on (changes in) physical activity level and determinant scores.

*Intervention plus *participants (n = 737), received the same tailored information as the basic intervention participants but additionally received tailored information about PA opportunities in their specific environment combined with access to a forum and e-buddy system on a website. The tailored environmental information included handouts with walking and cycling routes in their own neighbourhoods, examples of exercises to do at home, contact information for neighbourhood sports clubs that matched their interests and abilities, and map of their close neighbourhood on which walking and cycling possibilities were highlighted.

*Wait-list control participants *(n = 586) received nothing during the intervention period. They did receive one tailored letter, which was a combination of the three tailored letters from the basic tailored intervention, after completion of the research period.

### Questionnaires

Participants were asked to fill in a questionnaire at baseline, three months (during the intervention), and six months (two months post-intervention).

#### Demographics

At baseline, age, gender, weight and height (tot calculate BMI), highest completed educational level, employment and marital status were assessed.

#### Outcome measures

The primary outcome measures were total weekly days of PA behaviour, weekly minutes of walking, weekly minutes of cycling and weekly minutes of sports. The outcomes were assessed with the validated self-administrated Dutch Short Questionnaire to Assess Health Enhancing Physical Activity (SQUASH) at baseline and at six months. The reproducibility (r_spearman _= 0.58; 95%CI = 0.36-0.74) and relative validity (r_spearman _= 0.45; 95%CI = 0.17-0.66) of the SQUASH is reasonable[39]. *Total weekly days of PA *was measured with a self-reported single-item question: 'On how many days per week are you, in total, moderately physically active by undertaking, for example heavy walking, cycling, chores, gardening, sports or other physical activities for at least 30 minutes.' *Weekly minutes of walking, cycling and sports behaviours *were measured using the frequency (how many days per week), and duration (how many hours and minutes per day) of these activities.

##### Perceived environment

At baseline and three months, the perceived PA possibilities in people's direct environment were measured using an adapted version of the Neighbourhood Quality of Life Study Questionnaire [40]. The measure was found to have an acceptable to good reliability and acceptable validity[40]. The measure was constructed as the sum of eight conveniences of PA items each scoring 0 or 1: *i.e. For each of these places were you can exercise, please indicate if it is on a frequently travelled route or close to your work or home*. Followed by a list of 8 facilities: e.g. sports or fitness school, park or recreational ground, walking tracks, cycling tracks.

#### Process evaluation measure

At three and six months the use and appreciation of the tailored letters were measured. Use of the letters was measured in terms of whether the participants read (yes/no), saved (yes/no) and discussed (yes/no) the letters. Appreciation of the intervention was scored on a 5-point scale on how interesting, attractive, individualized, comprehensible, trustworthy, new the letters were, and whether they had the right quantity, and if the letters annoyed them (completely disagree [-2]; completely agree [+2]). Additionally, participants in the intervention *plus *condition were asked whether they used the information (i.e. walked/cycled routes, contacted sports club, visited website, did exercises, mapped out a walking/cycling route in their neighbourhood) and how interesting and motivating the environmental information was.

### Statistical analysis

An ANOVA examined baseline differences among the three conditions. Further analyses corrected for possible differences by including them as covariates into the regression analysis. Analyses were performed using SPSS for Windows (version 15.0).

Participants were nested within neighbourhoods within their municipality, with the probability of interdependence between the participants. To account for this interdependence, multilevel linear regression analyses with a random intercept for two levels (neighbourhood (2), individual (1)) were completed to analyze the efficacy of the interventions and mediation effects using MLWin (version 2.02). Outcome measures were the residual change scores of the level of minutes of walking, cycling and sports behaviour. Residual change scores can be calculated by regressing the outcomes at six months post-test onto their baseline values and represent the amount of change in PA independent of their baseline level. If possible differences between groups at baseline are not taken into account, the group with lower levels of PA are more likely by chance to increase their PA behaviour when compared to the group with higher levels of PA. Similarly, residual change scores were obtained for perceived PA possibilities in the environment at three and six months by regressing the score at three and six months respectively onto the baseline score.

#### Mediation analyses

To test the mediating effect of the change in perceived PA possibilities in the environment at three and six months on changes in the outcome measures at six months the product-of-coefficients test by MacKinnon was applied[41]. The test consists of 1) estimating the efficacy of the interventions in changing the outcome over six months (i.e. total PA, cycling, walking and sports) compared with the control condition (τ_Ibasic_C_; τ_Iplus_C_) and each other (τ_Iplus_Ibasic_)(*τ-coefficient*); 2) estimating the effect of the interventions on changes in the mediator (i.e. changes in the perceived PA possibilities over three months or six months) (*α-coefficient*); 3) estimating the association between the changes in the mediator and changes in the PA outcomes at six months (β-coefficient) when controlled for the intervention effect; 4) estimating the magnitude of the mediated effect over time, its statistical significance and the proportion mediated by calculating the product-of-coefficients by multiplying the α- and β-values (αβ-coefficient); and dividing it by its standard error (SEαβ = √(α^2 ^*SEβ^2^+β^2 ^*SEα^2^). The proportion mediated is estimated by dividing the mediated effect (αβ-coefficient) by the total intervention effect (*τ*' + αβ), in which *τ*' is the direct intervention effect when accounted for the indirect/mediated effect. The criteria of the mediation framework of MacKinnon suggests, in contrast to the mediation framework of Baron and Kenny, that potential mediating effects should also be analyzed even if the *τ*-coefficient (i.e. intervention effect on outcome) is not significant[41]. Therefore, in the current study, mediating analyses were also performed on behaviours which were not directly significantly influenced by the intervention. One of the requirements for conducting a full mediation analysis is the availability of a true control group. In this study, the three research conditions were recoded into two dummy variables in which the control group data was used as a comparison to assess the intervention effects on outcomes and mediators and to care for internal validity (i.e. control group as reference). All regression analyses included the significant personal characteristic (e.g. BMI, educational level, having a partner) as covariates.

#### Missing data

Logistic regression analyses, using SPSS (version 15.0) were conducted to examine if the drop-out rate was associated with baseline characteristics of the participants (i.e. age, gender, BMI, marital status, employment status, education level, baseline PA level and research condition). Participants who did not have a partner (Odds Ratio [OR]: 1.32; 95%CI: 1.0-1.7), those who had a higher BMI (OR: 1.03; 95%CI: 1.01-1.06), and those who were randomized in one of the intervention conditions (OR_Ibasic_: 2.15, 95%CI: 1.65-2.79; OR_Iplus_: 2.37, 95%CI: 1.84-3.05) were more likely to drop out of the study at six months. Subsequent analyses were corrected for BMI and marital status. According to Carpenter et al. [42], no objection for further analyses can be raised when drop-out is Missing at Random (MAR). Therefore additional analyses were conducted in order to assess whether the drop-out was MAR. It can be said that drop-out is MAR when a fully observed variable (e.g. research condition) is found, which defines groups within which data is Missing Completely At Random[42]. Hence, subsequent logistic regression analyses were conducted, in which it was examined whether the baseline characteristics of the participants (i.e. age, gender, BMI, marital status, employment status, education level, and baseline PA level) were significant predictors of the drop-out within each research group. Since no significant associations were found, we could conclude that the drop-out rate could be considered MAR, and that no objection could be raised for further analyses. Multilevel analyses have been shown to be very useful in handling missing data. Moreover, applying multilevel analyses to an incomplete dataset has been shown to be better than applying imputation methods[43]. Data were analyzed using the incomplete dataset including missing data (total data analyses) to account for the selective drop-out.

#### Process evaluation

Chi-square tests were used to test for differences between the two intervention conditions in use of the letters. Differences in appreciation of the tailored letters between the intervention conditions were analyzed with ANCOVA analyses, in which significant differences in baseline characteristics of the participants between the intervention conditions were included as covariates. Mediation analysis were conducted in order to identify whether differences in use and appreciation of the letters between the two interventions conditions, mediated the differences in effect between the two interventions following the method of MacKinnon[41].

## Results

### Participant characteristics at baseline

At baseline, 1971 older adults completed the questionnaire. At six months, 1411 completed the six-month questionnaire (response rate: 72%). Mean age was 64 years (SD = 8.6), 81% had a partner and the majority were women (57%). The mean BMI was 25.5 (SD = 3.8), 48.1% had low levels of education (primary, basic vocational or lower general school), and 47% were employed. The baseline characteristics of the participants are comparable with the general Dutch older adult population [5], indicating that the study sample was a good representative of the Dutch older adult population. Significant baseline differences were found for educational level, in which significantly more basic tailored intervention (51%) and control (50%) participants had lower levels of education than the intervention *plus *participants(42%; F (2,1928) = 5.0; p = 0.01).

At baseline, participants were on average moderately physically active for 4.1 (SD = 2.2) days per week for at least 30 minutes per day, walked on average 188.4 (SD = 201.4) minutes per week for recreation or transport, cycled on average 167.4 (SD = 199.5) minutes per week for recreation or transport, and participated in sports for 110.8 (SD = 155.1) minutes per week. As shown in table 1, at baseline participants in the basic tailored intervention cycled (Mean = 179.4; SD = 215.5) significantly more than the participants in the control condition (Mean = 147.5; SD = 174.3, F (2,1965) = 4.34; p < 0.05). No significant differences were found in outcome measures between the two intervention conditions. Participants perceived on average 4.5 (SD = 2.3) PA possibilities in their environment. At baseline, Intervention *plus *participants (Mean = 4.9; SD = 2.3), perceived significantly more PA possibilities in their environment than basic tailored intervention (Mean = 4.3; SD = 2.2) or control participants (Mean = 4.2; SD = 2.2; F(2,1965) = 21.3; p < 0.01). The use of residual change scores in all analyses corrected for these baseline differences.

**Table 1:** Baseline, post-test values, and intervention effects for each research condition on PA behaviours and perceived environment.

	Mean (SD)						Intervention effect (SE)		
	Baseline			Post-test			(τ-coefficient)		
	C	I_basic_	I_plus_	C	I_basic_	I_plus_	I_basic _vs C	I_plus _vs C	I_plus _vs I_basic_
**Total PA days/wk**	4.0 (2.1)	4.2 (2.2)	4.2 (2.2)	4.2 (2.0)	4.8 (2.0)	4.9 (2.0)	0.54 (0.13)***	0.51 (0.13)***	-0.0 (0.1)
**Cycling min/wk**	147.5 (174.3)	179.4 (215.5)	172.4 (202.5)	162.8 (192.3)	174.4 (206.0)	202.2 (214.7)	-11.0 (11.3)	**18.5 (11.0) ‡**	29.5 (11.2)**
**Walking min/wk**	164.7 (186.7)	189.2 (208.2)	173.0 (190.6)	176.4 (191.6)	201.0 (205.8)	188.0 (205.7)	11.1 (11.2)	1.2 (11.0)	-9.9 (11.2)
**Sports min/wk**	102.9 (145.7)	109.7 (158.6)	118.2 (158.9)	110.7 (153.6)	113.2 (154.3)	116.9 (155.1)	-5.2 (7.6)	-2.0 (7.5)	3.2 (7.6)
								**(α-coefficient)**	
**Perc. Environment** **_3 months_**	4.2 (2.2)	4.3 (2.2)	4.9 (2.3)	4.1 (2.2)	4.2 (2.3)	4.9 (2.3)	0.05 (0.16)	0.36 (0.15)*	0.31 (0.15)*
**Perc. Environment** **_6 months_**	4.2 (2.2)	4.3 (2.2)	4.9 (2.3)	4.1 (2.2)	4.6 (2.3)	5.3 (2.2)	0.06 (0.17)	0.51 (0.17)***	0.45 (0.17)***

* p < 0.05; ** p < 0.01; *** p < 0.001, ‡ p < 0.10; numbers in bold typeface: significant associations

PA: Physical Activity; C: Control condition; I_basic_: Basic tailored intervention condition; I_plus_: Intervention *plus *including environmental information; I_basic _vs C: effect basic tailored intervention compared with control condition; I_plus _vs C: effect intervention *plus *compared with control condition; I_plus _vs I_basic_: effect intervention *plus*compared with basic tailored intervention; Perc. Environment: number of perceived physical activity possibilities in direct environment [0-8]; SD: Standard deviation; SE: Standard Error. Regression models were adjusted for age, gender, educational status, occupational status, BMI, baseline levels of outcome, and within- district clustering effects.

### Intervention effects on PA behaviours (τ-coefficient)

As a first step in the mediation analyses, the intervention effect on the physical activities (i.e. total PA, cycling, walking and sports) were examined (see table 1). Both interventions were found to be effective in changing weekly days of PA (τ_Ibasic _= 0.54, SE = 0.13; p < 0.01; τ_Iplus _= 0.51, SE = 0.13; p < 0.01) when compared to the control condition. In addition, the intervention *plus *condition was found to be effective in changing cycling behaviour when compared to the control groups (τ_Iplus vs c _= 18.5, SE = 11.0; p = 0.09) and the basic tailored intervention group (τ_Iplus _vs τ_Ibasic_- 29.5, SE = 11.2; p < 0.01). However, the difference with the control condition was only borderline significant. No other significant differences in PA behaviour between the research conditions were found.

### Intervention effects on environmental perceptions (α-coefficient)

As a second step of mediation analyses, the effect of the interventions on the mediator was examined (see Table 1). The intervention *plus *condition was found to be more effective in positively changing the amount of perceived PA possibilities over three and six months than the control and basic tailored intervention condition. Specifically, at three months the intervention *plus *participants had increased their amount of perceived PA possibilities with 0.36 (SE = 0.15; p < 0.05) and 0.31 (SE = 0.15; p < 0.05) possibilities more than the control and basic tailored intervention group respectively when controlled for baseline values. At six months, intervention *plus *participants had further increased their amount of perceived PA possibilities with 0.51 (SE = 0.17; p < 0.001) and 0.45 (SE = 0.17; p < 0.001) when compared with the control and basic tailored intervention group. No difference in the change of perceived PA possibilities between the control and basic tailored intervention condition was found.

### Mediator effect on PA behaviour (β-coefficient)

As a third step of the mediation analyses, the effect of changes in the mediator on changes in the outcomes were examined (see Table 2 and 3). The results showed that the three months changes in perceived PA possibilities were positively associated with changes in weekly days of total PA (β = 0.05; SE = 0.03; p < 0.05), changes in weekly minutes of cycling (β = 7.27; SE = 2.68; p < 0.01), and changes in weekly minutes of sports (β = 9.63; SE = 1.92; p < 0.001) (see Table 2). No association was found between perceived PA possibilities and changes in walking behaviour (p = 0.20). Six months changes in perceived PA possibilities (see Table 3), were significantly associated with changes in weekly days of total PA (β = 0.06; SE = 0.03; p < 0.05) and changes in weekly minutes of sports (β = 6.23, SE = 1.87; p < 0.001). The association between perceived PA possibilities and cycling behaviour was no longer significant indicating that on the longer term the association between changes in perception and cycling behaviour had disappeared. Again, no association was found with walking behaviour.

**Table 2:** Mediated effects of changes in environmental perceptions over 3 months on changes in physical activity behaviours

	Effect environmental perceptions on outcome, β_3 months _(SE)	Mediated effect environmental perceptions, αβ_3 months _(SE)			
		Intervention *basic*	% ME	Intervention *plus*	% ME
**Total PA day/wk**	0.05 (0.03)*	0.00 (0.01)	-	**.02 (0.01)‡**	4.6
**Cycling min/wk**	7.26 (2.68)**	0.33 (1.13)	-	**2.62 (1.46) ‡**	14.5
**Walking min/wk**	-3.97 (2.60)	-0.18 (.63)	-	-1.43 (1.12)	-
**Sports min/wk**	9.63 (1.92)***	0.43 (1.49)	-	3.47 (1.61)*	>100

* p < 0.05; ** p < 0.01; *** p < 0.001, ‡ p < 0.10; numbers in bold typeface: significant associations

PA: Physical Activity; SE: Standard Error; %ME: proportion mediated. Regression models were adjusted for age, gender, educational status, occupational status, BMI, baseline levels of outcome, and within- district clustering effects.

**Table 3:** Mediated effects of changes in environmental perceptions over 6 months on changes in physical activity behaviours

	Effect environmental perceptions on outcome, β_6 months_ (SE)	Mediated effect environmental perceptions, αβ_6 months _(SE)			
		Intervention *basic*	% ME	Intervention *plus*	% ME
**Total PA day/wk**	0.06 (0.03)*	0.00 (0.01)	-	**0.03 (0.02)‡**	6.2%
**Cycling min/wk**	3.67 (2.51)	0.16 (0.53)	-	1.87 (1.38)	-
**Walking min/wk**	4.66 (2.97)	0.21 (0.70)	-	2.38 (1.66)	-
**Sports min/wk**	6.23 (1.87)***	0.29 (0.93)	-	3.17 (1.32)*	>100

* p < 0.05; ** p < 0.01; *** p < 0.001, ‡ p < 0.10; numbers in bold typeface: significant associations

PA: Physical Activity; SE: Standard Error; %ME: proportion mediated. Regression models were adjusted for age, gender, educational status, occupational status, BMI, baseline levels of outcome, and within- district clustering effects.

### Mediated effects (αβ-coefficient)

As a last step of the mediation analysis, the mediated effect and its significance were calculated. Three and six months changes in the perceived environment did not mediate the changes in any of the PA behaviours in the basic tailored intervention condition (see Table 2 and 3). In the intervention *plus *condition, 3.47 minutes (SE = 1.62; p < 0.05) and 3.17 minutes (SE = 1.32; p < 0.05) of change in sport, could be accounted by three and six months changes in environmental perceptions respectively. Further, the mediated effect of three and six months changes in environmental perceptions on changes in weekly days of total PA in the intervention *plus *condition showed a trend. These mediated effects accounted for 0.02 days per week (SE = 0.01; p = 0.09) and 0.03 days per week (SE = 0.02; p = 0.06) of changes in total PA behaviour. Three months changes in environmental perceptions mediated the changes in cycling behaviour and accounted for 2.62 minutes per week of changes in cycling behaviour (SE = 1.46; p = 0.07), although this association was borderline significant. The mediated effect of six months changes in environmental perceptions on changes in cycling behaviour was not significant, indicating that, changes in environmental perceptions exerted their effect on cycling behaviour only on the short term. No significant mediated effects were found on changes in walking behaviour (p = 0.20).

### Use and appreciation of the interventions

#### Use of the tailored letters

As shown in table 4, of the total population, 96% read the tailored letters, 68% saved the letters and 38% discussed the letters with others. When the two intervention conditions were compared, the letters of the intervention *plus *condition were saved more often than the tailored letters of the basic tailored intervention condition (χ^2 ^= 15.17; p < 0.01), however this difference did not mediate the intervention effect on any of the outcome variables.

**Table 4:** Use and Appreciation of the two computer-tailored interventions.

	Intervention_basic_ Number (%)	Intervention_plus_ Number (%)	χ^2^
**Use **(0-1)			
Read^b^	367/388 (95%)	400/418 (96%)	1.85
Saved^b^	215/355 (61%)	282/381 (74%)	15.17*
Discussed^b^	125/355 (35%)	151/381 (40%)	1.53
**Use environmental information **(0-1)			
Read	-	451/478 (94%)	
Contacted sports club	-	11/470 (2.3%)	
Visited website	-	52/470 (11%)	
Walked/Cycled trails^b^	-	161/393 (41%)	
Mapped out walking/cycling route^b^	-	57/393 (15%)	
Did exercises^b^	-	73/393 (19%)	

**Appreciation **(-2 to +2)	**Mean ± SD**	**Mean ± SD**	**F^a^**
Interesting	0.65 ± 0.72	0.85 ± 0.58	18.49**
Inviting	0.56 ± 0.75	0.80 ± 0.69	22.53**
Perceived individualization	0.26 ± 0.95	0.45 ± 0.86	11.36**
Comprehensible	0.98 ± 0.51	0.99 ± 0.46	0.00
Trustworthy	0.81 ± 0.58	0.93 ± 0.50	9.49**
Novelty	-0.34 ± 0.1.0	-0.32 ± 0.97	1.31
Right quantity	0.72 ± 0.64	0.75 ± 0.70	1.91
Irritating	-0.93 ± 0.87	-1.07 ± 0.95	4.62*
**Appreciation environmental information **(-2 to +2)			
Interesting^b^	-	0.74 ± 0.73	
Motivating^b^	-	-0.14 ± 0.94	

^a ^corrected for education level

^b ^measured with 6-months questionnaire

*p < 0.05; **p < 0.01; numbers in bold typeface: significant associations

#### Appreciation of the tailored letters

On average, 75% and 79% of the participants rated the letters of the basic tailored intervention and the intervention *plu*s respectively with a seven or higher (see table 4). When the appreciation of the two interventions were compared, the letters of the intervention *plus *were perceived as more interesting (F (1,636) = 18.5, p < 0.01), more inviting (F (1,645) = 22.5; p < 0.01), more individualized (F (1,643) = 11.4; p < 0.01), more trustworthy (F(1,643) = 9.49, p < 0.01) and less irritating (F (1,556) = 4.6; p < 0.05) when compared to the letters of the basic tailored intervention. The differences in appreciation between the two intervention, were not significantly associated with changes in PA behaviour and did therefore not mediate the intervention effects on any of the outcome variables.

#### Use and appreciation of environmental information

Among the intervention *plus *participants, 94% had read the environmental information, of them 71% agreed that the information was interesting, and 25% agreed that they got motivated by the information. Although 41% of the participants walked or cycled one or more of the provided existing walking or cycling trials, use of the other environmental information features was low. Only 19% did the provided exercises, 15% mapped out a walking or cycling route in their neighbourhood, 11% visited the website and only 2% of the participants contacted a sports club as a results of the contact information we provided.

## Discussion

This paper addresses the working mechanisms of an environmentally computer tailored PA intervention for older adults compared with a basic tailored intervention without environmental information. Based on the results of this study three conclusions can be drawn.

First, our results indicated that environmental perceptions is a relevant determinant of PA behaviour change. In addition, we found indications that providing environmental information is an effective intervention strategy aimed at changing PA behaviour. To specify, we found that changes in total weekly days of PA, minutes of cycling and minutes sport behaviour induced by the environmentally tailored intervention were mediated by changes in environmental perceptions. The computer tailored interventions were developed based on the assumption that they could be effective in changing PA behaviour through changes in the determinants that precede the behaviour. By adding environmental information to the computer tailored intervention (i.e. intervention *plus *condition), it was tried to change the perceptions of PA possibilities in order to influence PA behaviour. Results showed that the intervention *plus *condition succeeded in changing the perception of PA possibilities in the environment over three and six months. This in turn resulted in positive changes of total weekly days of PA, and minutes of cycling and sports behaviour, although the changes in minutes of cycling was only induced by the short-term changes in environmental perceptions. The mediated effects were partial, indicating that the intervention also had an effect on PA behaviour via other mediating mechanisms. Our results confirm the findings of Ries and colleagues [44] who found that two PA trials (i.e. Project STRIDE and Step into Motion) resulted in increased perceptions of available physical activity facilities among all research conditions. In addition, the authors found that these changes in environmental perceptions were associated with increases in weekly minutes of moderate and vigorous PA. Ries et al., however, were not able to find differences between the research conditions and did not conduct a mediation analysis [44]. Our current study verifies the importance of environmental perceptions as determinants of changing PA behaviour. Additionally, our study adds new understanding to the current knowledge on environmental PA interventions by presenting environmental perceptions as a mediator of PA interventions. This confirms that adding environmental information to a computer tailored intervention is an effective intervention strategy in changing physical activity behaviour among older adults.

Second, our results indicated that adding environmental information to tailored intervention mainly exerts its effect on cycling behaviour of older adults. Our results confirm the findings of the study of Ferney et al [28], who found that the implementation of an environmentally tailored PA website resulted in increased PA levels when compared to a motivation-only website. However, it must be noted that despite a significant mediated effect of environmental perceptions, providing environmental information did not result in an additional intervention effect on weekly days of PA and sports behaviour. It is possible that the type of information we provided is especially relevant for increasing active commuting, such as cycling behaviour, especially given the kind of environmental information that we provided. This could be confirmed by a recent literature review of Wendel-Vos et al. [45] who found that environmental perceptions were more important for commuting activities than for other PA behaviours. The authors found convincing evidence that perceived neighbourhood characteristics such as the connectivity of trials were associated with active commuting. Sports behaviour, on the other hand, was found to be convincingly associated with the availability of PA equipment at home, and less associated with perceived neighbourhood characteristics[45]. Since, it is likely that other environmental determinants (i.e. other than perceived PA possibilities) are relevant in changing sports behaviour [45-47], future interventions should provide other environmental information in order to target sports behaviour. Additionally, it is possible that non-environmental determinants (i.e. psychosocial variables) are more important in influencing sports behaviour than environmental determinants [22,48]. A multiple mediation model, including the mediated effects of both environmental perceptions and cognitive variables, was out of the scope of this study. Follow-up research should however analyze these mediating mechanisms and should examine if they differed between the two intervention conditions and different physical activities.

Third, we found that adding environmental information to an intervention resulted in more positive evaluations of the letters. Differences were observed in the use and appreciation of the tailored letters, mainly in favour of the tailored letters of the intervention *plus*. Our results confirm the findings of Ferney et al [28], who found that the participants of an environmentally tailored PA website reported higher levels of engagement than participants of a motivation-only website. Our enhanced evaluations could, according to the Elaboration Likelihood Model[49], have resulted in increased active information processing, resulting on its turn in more thoughtfully and profoundly processing and long-term behaviour change[49]. Although higher use and appreciation of the letters was expected to be important in successfully changing PA behaviour, none of the process evaluation scores mediated the intervention effect on any of the behavioural outcomes. This is in contradiction with the findings of Oenema et al [50] who could identify personal relevance, individualization and interestingness as mediators of the intervention effect of a tailored nutrition intervention compared with a generic nutrition information control group. The lack of mediated effect in our study could be explained by a lack of power. Although 925 intervention participants returned the six months questionnaire, which was consistent with our power calculations, only about 630 participants filled in the process evaluation questions. This might be due to the length of the questionnaire and the process evaluation questions being towards the end of it. Active information processing is a prerequisite in stably changing determinants and behaviour [51]. Hence, although we did not find a mediated effect, it is expected that the increased levels of active information processing in the intervention *plus *condition could result in increased levels of PA on the long term.

Based on our results suggestions for future intervention research can be made. First, the mediated effects we found were rather small, which could be explained by the fact that environmental information was not powerful enough, not visible or not perceived as interesting. This could be confirmed by the process evaluation data, which showed that only a small proportion of the participants visited the website, mapped out a route in the neighbourhood or contacted a sports clubs. Consequently, its influence on environmental perceptions could have been minimal. Future intervention research should therefore seek for other, more powerful, intervention strategies to provide environmental information. For example, by tailoring the environmental information to a more direct environment of the participant and by including some recent digital developments into the intervention (e.g. linking GIS with Google Earth). Second, in our study a dichotomous score was used as a measure of the perceptions of PA possibilities in the environment. However, as shown by recent cross-sectional studies [45-48], other environmental factors (e.g. connectivity, safety, aesthetics) might be important as well in changing PA behaviours. Future intervention research should therefore measure other perceptions of the environment and seek for intervention strategies in order to change these concepts.

Some limitations need to be addressed. First, the results relied on self-report. Therefore, the results might have been affected by response bias or over-reporting. By using validated questionnaires and giving all three research arms the same questionnaire, an attempt was made minimize bias. However, because it is possible that participation in an intervention influences the participants' interpretation of questionnaires, and in turn their response to questionnaires, the possibility of bias cannot be eliminated[52]. Second, the generalization of the results can be biased by the initial response and selective drop-out. Intervention participants were about twice as likely to drop out of the study as control participants. There are several possible reasons for this higher dropout rate. It is for example possible that participants who make fewer changes in PA because of the intervention often have less reason to stay in the program. Further, it is possible that because participants in the intervention conditions received their tailored advice at the start of the study, they are subsequently less reluctant to stay in the program and return questionnaires. Wait-list control participants, on the other hand, could have been more motivated to stay in the program since they had the prospect of receiving tailored advice at the end of the research period, while the participants in the intervention conditions only had the prospect of being eligible to win a prize. Higher drop-out rates among participants in the intervention condition have also been reported for other tailored interventions[50,53]. Twisk and De Vente found that when using multilevel analyses on datasets with high missing data, not imputing at all resulted in better analyses, with more accurate points of estimates, than any imputation method[43]. By analyzing the incomplete dataset including missing data using multilevel analyses, an attempt was made to account for selective drop-out. Third, differences in baseline values were found in educational level, environmental perception and minutes of cycling per week. The study accounted for this limitation by including educational level as a covariate in each regression analyses. In addition, the current study used residual change scores in the regression analysis to account for baseline differences in the outcome variables.

Bases on our results, we can conclude that changing the perception of PA possibilities in the environment is a relevant mediator of PA behaviour change. In addition, our study gave some first indications that providing environmental information in addition to a tailored intervention is an effective intervention strategy in order to change PA behaviour. To specify, adding environmental information to a computer tailored intervention resulted in increased environmental perceptions, increased minutes of cycling and more active information processing of the intervention. Although the environmental component needs further extension, adding environmental information to the tailored intervention was perceived as a valuable additional component of the intervention.

## List of Abbreviations

PA: Physical Activity.

## Competing interests

The authors declare that they have no competing interests.

## Authors' contributions

MVS, HDV, AM, CB and LL developed the concept and design of the study. MVS designed the interventions, coordinated the implementation of the interventions, data collection and analyses and wrote the original draft of the manuscript. HDV, AM, CB, and LL provided support during the development and implementation of the interventions, data collection and contributed to writing this manuscript. All authors have read and approved the final manuscript.
